# Impact of Different Attitudes toward Face-to-Face and Online Classes on Learning Outcomes in Japan

**DOI:** 10.3390/pharmacy11010016

**Published:** 2023-01-13

**Authors:** Mai Aoe, Seiji Esaki, Masahiro Ikejiri, Takuya Ito, Katsuhito Nagai, Yasutoshi Hatsuda, Yoshimi Hirokawa, Tomohisa Yasuhara, Takehiko Kenzaka, Toru Nishinaka

**Affiliations:** 1 Faculty of Pharmacy, Osaka Ohtani University, 3-11-1 Nishikiori-kita, Tondabayashi 584-8540, Osaka, Japan; 2 School of Pharmacy, Hyogo Medical University, 1-3-6 Minatojima, Chuo-ku, Kobe 650-8530, Hyogo, Japan; 3 School of Pharmacy, Wakayama Medical University, 25-1 Shicibancho, Wakayama 640-8156, Wakayama, Japan

**Keywords:** learning outcome, coronavirus disease 2019, online education

## Abstract

During the coronavirus disease 2019 (COVID-19) pandemic, online-based learning has become mainstream in many countries, and its learning outcomes have been evaluated. However, various studies have shown that online-based learning needs to be optimized in the future, and the number of reports for this purpose is currently not sufficient. The purpose in this study was to determine the relationship between academic performance and attitudes toward face-to-face and remote formats among Japanese pharmacy students enrolled in a course designed for knowledge acquisition. A combination of face-to-face and remote formats was used in a practice course for sixth-year pharmacy students, designed to improve academic performance through knowledge acquisition. To evaluate learning outcomes, we used a questionnaire that was administered to the course participants and the results of examinations conducted before and after the course. Online-oriented and face-to-face-oriented groups differed in their attitudes toward the ease of asking questions of faculty and communicating with the faculty members and classmates in each format. In a knowledge acquisition course for Japanese pharmacy students, the study revealed that the same academic outcomes were achieved, regardless of the students’ own perceptions of their aptitude for face-to-face or remote learning style.

## 1. Introduction

The impact of the novel coronavirus disease, COVID-19, has caused many changes in pharmacy education worldwide. Before the COVID-19 pandemic, face-to-face learning was the norm and online-based learning was complementary in most countries [1,2]. Since the pandemic, online-based learning has become mainstream in many countries, and its learning outcomes have been evaluated [3,4,5,6,7,8,9]. A scoping review reported in 2022 aimed to summarize the findings on the impact of COVID-19 on pharmacy education and the effectiveness of the real-world response measures [10]. However, while these reports indicate that online-based learning needs to be optimized in the future [3], the number of reports required for this purpose is not sufficient.

Pharmaceutical education, which is the route to becoming a pharmacist in Japan, is a six-year system that includes 22 weeks of practical internship in the fifth year. Most sixth-year students will perform their graduation research and study for the national pharmacist examination to be held at the end of the school year. Before the COVID-19 pandemic, traditional face-to-face learning was also the norm in Japan. However, from the 2020 academic year, Japan’s Ministry of Education, Culture, Sports, Science and Technology (MEXT) has asked academies to promote the use of a variety of remote learning formats and methods with maximum credit limit to prevent further outbreaks of COVID-19 [11]. This was the beginning of the full-scale introduction of online learning in pharmacy education in Japan.

Our university is no exception, and has decided to use remote learning in addition to the face-to-face learning that has been offered in the past. In the 2020 academic year, regarding the practice course for sixth-year students, few face-to-face formats were held, and video-on-demand, prepared in advance by faculty members, were distributed. However, many students complained that it was difficult to establish a rhythm to their daily lives. We were also able to extract the advantages of video-on-demand, such as the ability to watch repeatedly in our own time. However, we prioritized the rhythm of their daily lives. On the other hand, some students had concerns about being infected with COVID-19 during the face-to-face learning classes, and preferred online-based learning. Therefore, in the 2021 academic year, it was decided that a combination of face-to-face and online learning formats would be offered.

Various reports have evaluated online-based learning during the COVID-19 pandemic for pharmacy education in Japan [12,13,14]. However, to our knowledge, the relationship between perceptions toward face-to-face or remote learning formats and academic performance in a practice course for knowledge acquisition has not been clarified. Therefore, this study aimed to clarify the perceptions and academic performance of pharmacy students in Japan toward face-to-face and online learning formats in a practice course for knowledge acquisition. This study is novel in that it shows a relationship between Japanese pharmacy students’ attitudes toward face-to-face and remote learning formats and their academic performance. This study can contribute to the accumulation of knowledge regarding the optimization of online-based learning.

## 2. Materials and Methods

### 2.1. Practice Course in This Study

The practice course was delivered for sixth-year pharmacy students at a private university in the 2021 academic year. The course offered both face-to-face and online attendance, with the aim of improving academic performance through knowledge acquisition. A summary of the practice course is shown in Table 1. This course covered the subjects studied up to the sixth year: physics, chemistry, biology, hygienic pharmacy, pharmacology, pathophysiology pharmacotherapeutics, pharmaceutics, pharmaceutical regulations and systems and pharmaceutical practices.

In this course, most of the faculty members used PowerPoint^®^ and lecture formats. Classes in this course consisted of lectures summarizing the main points and exercises in which students solved problems and later received the explanations; the breakdown of classes was left to the discretion of the faculty member. Students in the face-to-face learning group received their lectures in the same classroom as the faculty members.

The online format was delivered via Zoom^®^ at the same time as the face-to-face format. The class was not recorded, and students were not given the opportunity to view the video after class. Most of the classes were one-way, with little interactive engagement; however, questions could be asked using an online chat feature. The main difference between the face-to-face and online learning formats examined in this study was the difference in the form of questions to faculty and the form of communication with faculty members and students.

The students were permitted to choose whether to attend face-to-face or online classes, and were allowed to change their choice during the course. To evaluate the learning outcomes of the practice course, we used a questionnaire that was administered to the students and the results of examinations conducted before and after the practice course.

### 2.2. Survey of Online Format Tendency Level

A survey on the students’ perceptions of this practice course toward face-to-face and online learning formats was conducted in November 2021. The survey questionnaire consisted of six Likert-type questions, each with five choices: 5, agree; 4, somewhat agree; 3, neither; 2, not so much; 1, disagree. The students were asked to answer the following questions: Face-to-face format is suitable for myself (Q1); Online format is suitable for myself (Q2); It is easy to communicate with faculty or classmates in face-to-face format (Q3); It is easy to ask questions of faculty or classmates in face-to-face format (Q4); It is easy to communicate with faculty or classmates in online format (Q5); It is easy to ask questions of faculty or classmates in online format (Q6). The content ideas of the questionnaire were based on previously published studies involving academic courses delivered in face-to-face and remote learning formats [15,16,17].

To determine whether students’ impressions tended to be that online or face-to-face formats were more suited to them, the difference between the responses in Q2 and Q1 was calculated (Q2 response number minus Q1 response numbers). This value was defined as “online format tendency level.” Based on the results, we classified the respondents into three groups. Positive values in online format tendency level indicated that the participants were suited to remote format; this was classified as the “online format-oriented group.” A value of zero indicated that there is no difference in the student’s orientation toward face-to-face and online formats; this was classified as the “neither group.” Finally, negative values indicated that the participants were suited to face-to-face format; this was classified as the “face-to-face format-oriented group.”

### 2.3. Characteristics of Three Groups: Online Format-Oriented Group, Neither Group and Face-to-Face Format-Oriented Group

#### 2.3.1. Face-to-Face Format Ratio in Each Group

For each group categorized by online and face-to-face format-oriented attitudes, the face-to-face format ratio in this course was calculated as follows. In each student, the number of days to actually attend the face-to-face format class was divided by the number of total class days during the course. The face-to-face format ratio in each group was shown as the average of the individual ratio.

#### 2.3.2. Survey on the Students’ Perceptions toward Ease of Asking Questions of the Faculty Members and Communication with Faculty Members and Classmates in Face-to-Face and Remotely

To understand the characteristics of the three groups of students classified according to “online format tendency level,” the ease of asking questions and communicating in the face-to-face and remote format were confirmed using a questionnaire (Q3–6). The ease of asking questions of faculty members and communicating with faculty members and classmates in face-to-face and online were confirmed using a questionnaire.

### 2.4. Relationship between Online Format Tendency Level and Academic Performance

We examined the growth in academic performance of three groups in response to this course. We used the results of examinations conducted before and after the course. By comparing the percentage of correct answers on the examination before the course (in June 2021) and after the course (in November 2021) we evaluated the growth in academic performance resulting from the course.

Tukey’s honestly significant difference test was used to compare growth in academic performance between three groups. The Steel–Dwass test was used to compare the face-to-face format ratio between groups. Fisher’s exact test was used to compare questionnaire responses between groups. JMP Statistical Software (version 14.2. 0) was used for statistical analyses.

## 3. Results

Data from 121 (96.0%) of the 126 six-year students from the academic year 2021 were included in the study. We excluded four students who did not take the examination and one student who did not respond to the questionnaire, and no student refused to answer.

### 3.1. Online Format Tendency Level

**Figure 1:**
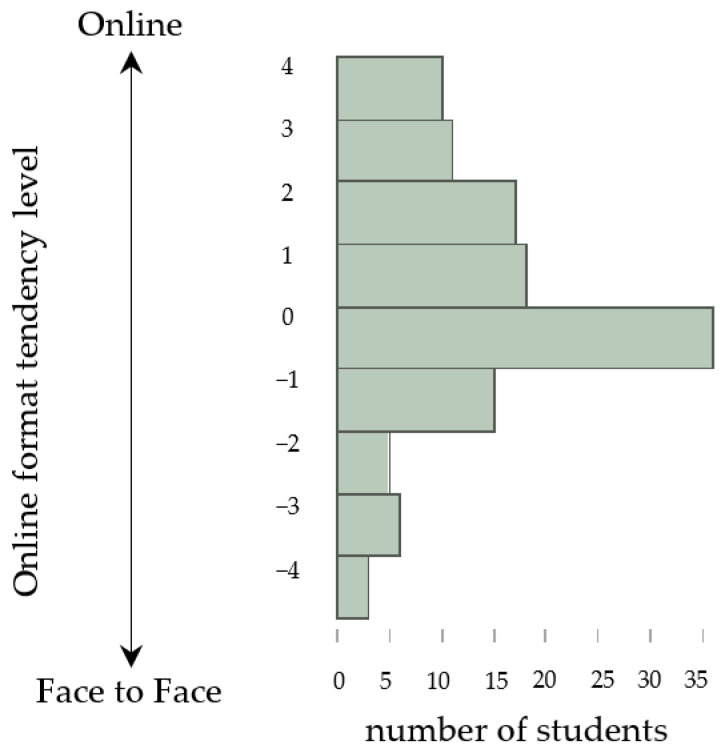
Online format tendency level and number of students.

The results of the questionnaire (Q1 and Q2) are shown in Table 2. Online format tendency levels (Q2 response numbers minus Q1 response numbers) are shown in Figure 1. The average of the online format tendency level of all students was 0.58 ± 1.92. There were 29 students in the face-to-face format-oriented group (24.0%), 36 students in the neither group (29.8%), and 56 students in the online format-oriented group (46.3%).

### 3.2. Characteristics of Each Group

#### 3.2.1. Face-to-Face Format Ratio

The mean face-to-face format ratio in each group during the practice course was 57.1 ± 3.6% for the face-to-face format-oriented group, 21.9 ± 3.3% for the neither group and the 10.9 ± 2.6% for the online format-oriented group. The face-to-face format-oriented groups tended to have a higher face-to-face format ratio. A comparison of the face-to-face format ratio for each group indicated a statistically significant difference between the groups in the comparisons of the face-to-face format-oriented group versus the neither group (*p* = 0.0050), the online format-oriented group versus the face-to-face format-oriented group (*p* < 0.001) and the neither group versus the online format-oriented group (*p* < 0.001). The online format-oriented groups had lower face-to-face format ratio (i.e., higher online format ratio) than the other groups.

There were cases where there was no positive correlation between the awareness tendency and attendance rate in the face-to-face format. For example, some students in the online format-oriented group tended to have higher face-to-face attendance rates. However, the number of samples was small (less than 5). Therefore, this study did not investigate the difference in academic performance in these cases.

#### 3.2.2. Results of the Survey of Students’ Perceptions toward the Ease of Asking Questions of Faculty Members and Communication with Faculty Members or Classmates in Face-to-Face and Online Formats

The results of the questionnaire regarding the ease of asking questions of faculty members and communication with faculty members or classmates are shown in Table 3 and Table 4. The online format-oriented group had significantly lower average values for the ease of asking questions and the ease of communication in face-to-face format (Q3,4) than those of the face-to-face format-oriented group (*p* = 0.010, *p* = 0.041). The online format-oriented group tended to have higher average values for the ease of asking questions in the online format (Q5 and Q6) than the face-to-face format-oriented group, although the differences were not significant (*p* = 0.322, *p* = 0.083). The ease of questioning of faculty members and communication with faculty members and classmates correlated with the preference for face-to-face or online formats (r = 0.20, *p* < 0.05).

### 3.3. Growth of Academic Performance

The mean academic performance of all students in the pre- and post-course examinations shown as the percentage of correct answers were 43.21 ± 9.58% and 51.07 ± 9.94%, respectively. The mean growth between the pre- and the post-course examination was 7.86 ± 6.54.

**Figure 2:**
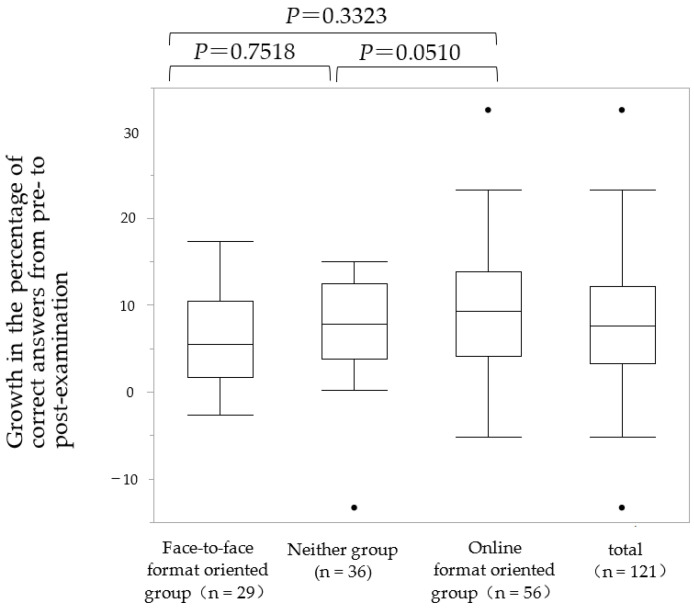
Growth in academic performance of the three groups by online format tendency level.

The percentage of growth in academic performance between the pre- and the post-course examinations for each group is shown in Figure 2. The mean growth of the face-to-face format-oriented group was 7.24 ± 1.20, and that of the online format-oriented group was 9.33 ± 0.86. The *p* value between groups was 0.3323, and did not indicate a significant difference. The growth in the examination results for the neither group was 6.08 ± 1.07. When comparing the performance of the neither group and the online format-oriented group in the examination, the growth in the examination was higher for the online format-oriented group, and the *p* value was 0.0510.

## 4. Discussion

The most significant finding of this study is that Japanese pharmacy students achieved the same academic performance in a practice course independent of the students’ perceptions toward face-to-face or online learning formats.

Prior studies have reported little difference in examination scores between actual face-to-face and online classes [17,18,19,20,21,22]. The results of this study are novel in that they reveal that the learning outcomes were equivalent, regardless of whether the individual was face-to-face or online oriented. In contrast, in terms of academic performance outcomes, an online learning format was reported to be superior to face-to-face learning for Korean pharmacy students [4]. The reason for the difference in the results was that the video could be viewed repeatedly, whereas, in this study, the video was not available.

In this survey, the number of students in the face-to-face format-oriented group was smaller than that of the online format-oriented group (Table 3). Studies conducted prior to the COVID-19 pandemic reported that approximately half of pharmacy students preferred online education to face-to-face education [23]. During the COVID-19 pandemic, reports in other countries have also shown a preference for online education compared to face-to-face education [4,5]. These findings suggest that since the onset of the COVID-19 pandemic, the number of students who prefer online-based learning has increased. However, neither group constituted 29.8% of total students. Furthermore, the online-oriented group tended to have higher academic performance than the neither group (Figure 2). Students’ ability to self-regulate their own learning is a crucial factor in their learning success. It has been reported that online-based education is more likely to offer opportunities for promoting self-regulated learning [24,25]. Therefore, it is conceivable that the online-oriented group had more established self-regulated learning than the neither group, leading to better academic performance.

The ease of questioning of faculty members and communication with faculty members and classmates correlated with the preference for face-to-face or online formats. The online-oriented and face-to-face–oriented groups differed in their attitudes toward the ease of asking questions of faculty members and communicating with faculty members and classmates in face-to-face and online formats (Table 3). However, we did not assess or specify why students preferred face-to face or online format. However, other studies have found that “greater flexibility” and “self-paced learning” are important reasons for the students to prefer the online format [16,26,27]. Future studies should include an investigation of the reasons for the preference of Japanese pharmacy students in format selection and other external factors that influence their academic performance.

Previous studies have shown that the adoption of online format courses in higher education remains challenging for several reasons, including dealing with the approved educational platform, noise during live lectures and technical problems, such as livestream quality and unavailability of the internet [28,29,30,31]. We believe that this will help both educators and learners to determine a more effective learning style in future pharmacy education, after the end of the pandemic, from the viewpoint of perceptions toward face-to-face or remote learning formats.

We should be cautious about generalizing this study because of the limitation that the study was held at one private university pharmacy school in Japan. Therefore, the results of this study cannot be said to be representative of all Japanese pharmacy schools.

## 5. Conclusions

In a knowledge acquisition course for the Japanese pharmacy students, the study revealed that the same academic outcomes were achieved regardless of the students’ own perceptions of their aptitude for face-to-face or remote learning style. This study can contribute to the accumulation of knowledge regarding the optimization of online-based learning.

## Figures and Tables

**Table 1:** Flow of this course and evaluation methods.

Time	Number of Hours of Course	Evaluation Method
Before course (Early June)	―	Pre-exam
Term 1 (Mid-June to late July)	3 h × 38 classes	―
Term 2 (Late July to early September)	3 h × 37 classes	―
Term 3 (Early September to mid-October)	3 h × 36 classes	―
After course (Mid-November)	―	Post-exam

**Table 2:** Questionnaire items and simple tabulation results of the survey (Q1 and Q2).

Time	1: Disagree	2: Not so much	3: Neither	4: Somewhat agree	5: Agree
Q1: Face-to-face formats are suitable for myself	11	25	46	29	10
Q2: Online formats are suitable for myself	6	6	45	38	26

**Table 3:** Impressions of asking questions and communication in the three groups (result of survey Q3–Q6).

			1: Disagree	2: Not so much	3: Neither	4: Somewhat agree	5: Agree	Average ± SD
Total	Q3: Easy to communicate with faculty or classmates in face-to-face format		1	8	13	50	49	4.14 ± 0.94
	Q4: Easy to ask questions of faculty or classmates in face-to-face format		2	10	17	43	49	4.05 ± 0.87
	Q5: Easy to communicate with faculty or classmates in online format		28	54	33	5	1	2.15 ± 0.40
	Q6: Easy to ask questions of faculty or classmates in online format		22	42	36	17	4	2.50 ± 0.32
Face-to-face format-oriented group (*n* = 29)		Q3	0	0	0	11	18	4.62 ± 1.38
		Q4	0	0	2	10	17	4.52 ± 1.27
		Q5	11	11	7	0	0	1.86 ± 0.37
		Q6	10	12	6	1	0	1.93 ± 0.34
Neither group (*n* = 36)		Q3	1	1	6	16	12	4.03 ± 0.86
		Q4	2	1	7	14	12	3.92 ± 0.79
		Q5	5	17	11	2	1	2.36 ± 0.42
		Q6	5	9	12	9	1	2.78 ± 0.43
Online format-oriented group (*n* = 56)		Q3	0	7	7	23	19	3.96 ± 0.81
		Q4	0	9	8	19	20	3.89 ± 0.76
		Q5	12	26	15	3	0	2.16 ± 0.41
		Q6	7	21	18	7	3	2.61 ± 0.34

**Table 4:** Fisher’s exact test results (*p* value).

	Q3	Q4	Q5	Q6
Face-to-face oriented group vs. Neither group	0.021	0.142	0.132	0.019
Neither group vs. Online oriented group	0.337	0.121	0.751	0.513
Face-to-face oriented group vs. Online oriented group	0.010	0.041	0.322	0.083

## Data Availability

Data is available from the corresponding author upon reasonable request.

## References

[1] Costa-Santos C., Coutinho A., Cruz-Correia R., Ferreira A., Costa-Pereira A. (2007). E-learning at porto faculty of medicine. A case study for the subject ‘Introduction to Medicine’. Stud. Health Technol. Inform..

[2] Tagg P.I., Arreola R.A. (1996). Earning a master’s of science in nursing through distance education. J. Prof. Nurs. Off. J. Am. Assoc. Coll. Nurs..

[3] Carla P. (2022). Perceptions of pharmacy students on the E-learning strategies adopted during the COVID-19 pandemic: A systematic review. Pharmacy.

[4] Yoo H., Kim D., Lee Y.M., Rhyu I.J. (2021). Adaptations in anatomy education during COVID-19. J. Korean Med. Sci..

[5] Al-Alami Z.M., Adwan S.W., Alsous M. (2021). Remote learning during COVID-19 lockdown: A study on anatomy and histology education for pharmacy students in Jordan. Anat. Sci. Educ..

[6] Chen T., Peng L., Yin X., Rong J., Yang J., Cong G. (2020). Analysis of user satisfaction with online education platforms in China during the COVID-19 pandemic. Healthcare.

[7] Shawaqfeh M.S., Al Bekairy A.M., Al-Azayzih A., Alkatheri A.A., Qandil A.M., Obaidat A.A., Al Harbi S., Muflih S.M. (2020). Pharmacy students perceptions of their distance online learning experience during the COVID-19 pandemic: A cross-sectional survey study. J. Med. Educ. Curr. Dev..

[8] Attarabeen O.F., Gresham-Dolby C., Broedel-Zaugg K. (2021). Pharmacy student stress with transition to online education during the COVID-19 pandemic. Curr. Pharm. Teach. Learn..

[9] Memon I., Feroz Z., Alkushi A., Qamar N., Ismail F. (2021). Switching from face-to-face to an online teaching strategy: How anatomy and physiology teaching transformed post-COVID-19 for a university preprofessional program. Adv. Physiol. Educ..

[10] Courtney J., Titus-Lay E., Malhotra A., Nehira J., Mohamed I., Mente W., Uyen L., Buckley L., Feng X., Vinall R. (2022). COVID-19-driven improvements and innovations in pharmacy education: A scoping review. Pharmacy.

[11] Ministry of Education, Culture, Sports, Science and Technology (2020). Notification: Commencement of Classes, etc. at Universities, etc. in fiscal 2020. https://www.mext.go.jp/content/20200324-mxt_kouhou01-000004520_4.pdf.

[12] Tanaka S., Kato R., Kobayashi Y., Kobayashi A., Yamamoto H. (2021). Holding online classes for the Advanced Clinical Psychology. Jpn. J. Pharm. Educ..

[13] Yanagita T., Nakamura T., Yoshikawa T. (2021). A trial of practical pharmacotherapy education using online role-play in Medical, Pharmaceutical and Dental education. Jpn. J. Pharm. Educ..

[14] Yasuhara T., Kushihata T., Ueda M., Kurio W., Sone T. (2021). Education for freshmen in the faculty of pharmacy in 2020. Jpn. J. Pharm. Educ..

[15] Freeman M.K., Schrimsher R.H., Kendrach M.G. (2006). Student perceptions of online lectures and WebCT in an introductory drug information course. Am. J. Pharm. Educ..

[16] Rochester C.D., Pradel F. (2008). Students’ perceptions and satisfaction with a web-based human nutrition course. Am. J. Pharm. Educ..

[17] Porter A.L., Pitterle M.E., Hayney M.S. (2014). Comparison of online versus classroom delivery of an immunization elective course. Am. J. Pharm. Educ..

[18] Woo M.A., Kimmick J.V. (2000). Comparison of internet versus lecture instructional methods for teaching nursing research. J. Prof. Nurs..

[19] Buckley K.M. (2003). Evaluation of classroom-based, web-enhanced, and web-based distance learning nutrition courses for undergraduate nursing. J. Nurs. Educ..

[20] Ryan G., Lyon P., Kuma K., Bell J., Barnet S., Shaw T. (2007). Online CME: An effective alternative to face-to-face delivery. Med. Teach..

[21] Summers J.J., Waigandt A., Whittaker T.A. (2005). A comparison of student achievement and satisfaction in an online versus a traditional face-to-face statistics class. Innov. High Educ..

[22] Faulkner T.P., Christoff J.J., Sweeney M.A., Oliver N. (2005). Pilot study of a distance-learning methodology used on campus for first professional degree pharmacy students in an integrated therapeutics module. Am. J. Pharm. Educ..

[23] Mohammed S.K., Sarath C.C., Shabeer A.T., Dilip C., Naseef P.P., Mamdouh M.A.A., Hafees M. (2022). Pharmacy student’s challenges in virtual learning system during the second COVID 19 wave in southern India. Soc. Sci. Humanit. Open.

[24] Roddy C., Amiet D.L., Chung J., Holt C., Shaw L., McKenzie S., Garivaldis F., Lodge J.M., Mundy M.E. (2017). Applying best practice online learning, teaching, and support to intensive online environments: An integrative review. Front. Educ..

[25] Wong J., Baars M., Davis D., Van Der Zee T., Houben G.J., Paas F. (2019). Supporting self-regulated learning in online learning environments and MOOCs: A systematic review. Int. J. Hum. Comput. Interact..

[26] Thwin E.P. (2017). Practical tips for effective and efficient anatomy teaching. Med. Sci. Educ..

[27] Kelsey A.H., McCulloch V., Gillingwater T.H., Findlater G.S., Paxton J.Z. (2020). Anatomical sciences at the University of Edinburgh: Initial experiences of teaching anatomy online. Transl. Res. Anat..

[28] Lange C., Costley J. (2020). Improving online video lectures: Learning challenges created by media. Int. J. Educ. Technol. High Educ..

[29] Mawarni I.T., Ratnasari N., Handayani A.N., Muladi M., Wibowo E.P., Untari R.S. (2020). Effectiveness of WhatsApp in improving student learning interests during the COVID-19 pandemic. Proceedings of the 4th International Conference on Vocational Education and Training (ICOVET 2020).

[30] Alkhawaja M.I., Abd Halim M.S. (2019). Challenges of e-learning system adoption in Jordan higher education. Int. J. Acad. Res. Bus. Soc. Sci..

[31] Salas-Pilco S.Z., Yuqin Y., Zhe Z. (2022). Student engagement in online learning in Latin American higher education during the COVID-19 pandemic: A systematic review. Br. J. Educ. Technol..

